# Partial breast reconstruction of 30 cases with peri-mammary artery perforator flaps

**DOI:** 10.1186/s12893-023-01937-4

**Published:** 2023-02-21

**Authors:** Meiying Shen, Yuhang Xu, Zongyuan Wu, Jiaming Wang, Huawen Pan, Bo Xu

**Affiliations:** 1 Jinan University, Guangzhou, 510000 China; 2 Mammary Gland, Maoming People’s Hospital, Maoming, 525000 China

**Keywords:** Oncoplastic breast-conserving surgery, Perforator flap, Breast partial reconstruction satisfaction

## Abstract

**Background:**

Volume replacement is one of the vital techniques of oncoplastic surgery (OPS) when applying breast-conserving surgery. The clinical application of peri-mammary artery perforator flaps for this indication is uneven in China. Here, we describe the results of our clinical experience with peri-mammary artery flaps for partial breast reconstruction.

**Methods:**

In this study, 30 patients underwent partial breast resection for quadrant breast cancer followed by partial breast reconstruction with peri-mammary artery perforator flaps, which included the thoracodorsal artery perforator flap (TDAP), anterior intercostal artery perforator flap (AICAP), lateral intercostal artery perforator flap (LICAP), and lateral thoracic artery perforator flap (LTAP). All the patients’ operation plans were discussed comprehensively and were performed by sticking to every step. The satisfaction outcome was assessed with the extracted version of the BREAST-Q version 2.0, Breast Conserving Therapy Module Preoperative and Postoperative Scales both preoperatively and postoperatively.

**Results:**

According to the study outcomes, the mean flap size was 5.3*4.2*2.8 cm (3.0–7.0*3.0–5.0*1.0–3.5 cm). The mean surgical time was 142 min (100–250 min). No partial flap failure was detected, and no severe complications were observed. Most patients were satisfied with the outcomes regarding the dressing, sexual life, and breast shape postoperation. Furthermore, the sensation of the surgical area, scar satisfaction, and recovery state gradually improved. Overall, LICAP and AICAP had higher scores when different flaps were compared.

**Conclusions:**

Based on this study, we found that peri-mammary artery flaps had significant value in breast-conserving surgery, especially in patients with small or medium-sized breasts. Perforators could be detected by vascular ultrasound before the operation. More than one perforator could be found most of the time. No severe complications occurred when performing a suitable plan, including discussing and recording the operation procedure; the focus of care, the choice for precise and proper perforators, and the mechanism for hiding the scars were all considered and recorded in a specific chart. Patients were satisfied with the reconstruction technique of peri-mammary artery perforator flaps after breast-conserving, and the satisfaction of AICAP and LICAP was higher. In general, this technique is suitable for partial breast reconstruction and has no negative impact on patient satisfaction.

## Introduction

Breast-conserving surgery has become the standard treatment for early-stage breast cancer. Breast preservation surgery and radiotherapy have the same local recurrence and survival rates as total breast resection [1, 2]. However, routine breast preservation surgery will result in postoperative local depression, poor breast appearance, and even abnormal problems [3–5]. Oncoplastic breast-conserving surgery (OBCS) combines surgical oncology and plastic surgery techniques. Applying this technique is considered to increase the chances of breast conservation and reduce the rate of positive margins and the proportion of surgical reresection, improving the appearance of the breast at the same time [6]. The OBCS technique is mainly divided into volume displacement and volume replacement. When breast preservation defects reach more than 20% of the breast, solely relying on volume displacement will often result in a poor appearance. Volume replacement is required for a certain proportion of breast volume defects or with a particular tumour location [7]. Currently, most specialized breast centres are mature in using the latissimus dorsi flap or lateral adipofascia flap for volume replacement in China. However, the need to sacrifice part or all of the latissimus dorsi muscle or the range of movement after fascia flap acquisition is minimal. Based on this background, this study summarizes the operation procedure and clinical effects of the application of partial breast reconstruction with the peripheral breast artery in patients from the centre. This study provides experience and data for the further application of this technique in China.

## Method

From January 2021 to March 2022, the Breast Department of Maoming People’s Hospital, Guangdong Province, completed partial breast reconstructions after breast conservation surgery with lateral chest wall fascia flaps, which were performed by the same surgeon. The surgical methods and postoperative results of the 30 patients were summarized and analysed. (see Table 1 for the basic information of the patients).

**Table 1:** Patients’ basic information

Patient	Date	Age (y)	Breast size	Tumour size	Location	Resection size	Flap	Surgery time (min)	Follow-up (mths)	Complication	Complementary treatment
1	2021-7	65	B	2.0 cm*2.5 cm	Upper outer of left side	3.5*3.0*2.5 cm	6.0*3.0*2.0 cm (TDAP)	180	11	No	HT + CMT + RDT
2	2021-2	39	A	2.0 cm*2.0 cm	Upper inner of left side	3.0*2.5*2.0 cm	6.0*3.0*2.0 cm (AICAP)	250	11	Fat necrosis	HT + CMT + RDT
3	2021-11	51	C	1.0 cm*0.5 cm	Upper outer of right side	3.0*2.0*2.0 cm	4.0*3.0*3.0 cm (Z型)	120	7	No	HT + RDT
4	2021-11	56	A	1.3 cm*0.9 cm	Upper outer of left side	2.5*2.0*1.5 cm	4.0*3.0*2.0 cm (LTAP)	115	7	No	CMT + RDT
5	2021-11	51	B	2.9 cm*2.0 cm	Lower inner of right side	4.0*3.0*2.0 cm	6.0*3.0*2.0 cm (AICAP)	145	7	No	CMT + RDT
6	2021-1	48	B	3.1 cm*2.5 cm	Nipple lower of right side	4.0*4.0*2.0 cm	3.5*3.0*3.0 cm (Z型)	160	17	No	HT + CMT + RDT
7	2021-11	50	A	3.0 cm*2.0 cm	Upper outer of right side	4.0*4.0*3.0 cm	3.0*2.0*2.0 cm (LTAP)	105	7	No	HT + CMT + RDT
8	2022-1	46	C	4.1 cm*3.0 cm	Upper outer of left side	6.0*5.0*3.5 cm	7.0 cm*5.0**3.0 cm (LICAP)	190	5	No	HT + CMT + RDT
9	2022-2	65	C	2.4 cm*2.5 cm	Lower outer of left side	4.5*4.5*3.0 cm	6.0 cm*5.0*3.0 cm (LICAP)	165	4	Venous congestion	HT + CMT + RDT + TT
10	2021-8	33	B	1.3 cm*1.0 cm	Lower outer of left side	2.5*2.0*2.0 cm	6.0*3.0*2.0 cm (AICAP)	128	10	No	CMT + RDT
11	2021-11	60	B	2.5 cm*1.5 cm	Upper outer of left side	4.0*3.0*3.0 cm	6.0*4.0*2.0 cm (LTAP)	126	7	No	HT + CMT + RDT
12	2022-1	56	B	1.5 cm*1.0 cm	Upper outer of left side	3.0*2.0*2.0 cm	4.0*3.0*3.0 cm (LICAP)	110	5	No	HT + RDT
13	2021-10	53	B	2.0 cm*1.5 cm	Upper outer of right side	3.0*2.0*2.0 cm	4.0*3.0*1.5 cm (LICAP)	118	8	No	HT + CMT + RDT
14	2021-3	45	B	1.5*1.5 cm	Lateral outer of left side	3.0*2.0*2.0 cm	3.5*3.0*3.1 cm (TDAP)	165	15	No	HT + RDT
15	2021-12	38	A	2.0 cm*2.1 cm	Upper outer of left side	3.0*3.0*2.0 cm	3.0*2.0*2.1 cm (LICAP)	114	6	No	CMT + RDT
16	2021-3	50	C	2.5 cm*2.6 cm	Lower outer of left side	3.5*3.0*2.5 cm	5.0 cm*4.0*3.0 cm (AICAP)	155	15	No	CMT + RDT
17	2021-4	45	B	1.3 cm*1.1 cm	Lower of left side	2.5*2.0*2.0 cm	3.0 cm*3.0*2.0 cm (AICAP)	164	14	No	HT + RDT
18	2021-4	55	B	2.5 cm*1.6 cm	Upper outer of left side	3.5*2.0*2.0 cm	5.0*3.0*2.0 cm (LICAP)	172	14	No	HT + CMT + RDT + TT
19	2021-6	58	A	1.5 cm*1.1 cm	Upper inner of right side	2.5*2.0*2.0 cm	5.0*3.0*2.0 cm (AICAP)	145	12	No	HT + RDT
20	2021-5	35	B	2.0 cm*1.6 cm	Upper outer of right side	3.0*3.0*1.5 cm	4.0*3.0*2.0 cm (LICAP)	210	13	No	HT + CMT + RDT
21	2022-2	33	B	1.5*1.6 cm	Lower inner of left side	2.5*2.0*1.5 cm	5.0*3.0*1.0 cm (AICAP)	105	4	No	CMT + RDT + TT
22	2021-7	46	C	4.0 cm*3.2 cm	Lateral outer of right side	5.0*3.5*2.5 cm	6.5*4.0*3.5 cm (TDAP)	207	11	No	CMT + RDT
23	2022-1	48	B	2.5 cm*2.7 cm	Lower of right side	3.5*3.5*2.0 cm	5.0*2.0*1.5 cm (AICAP)	103	6	No	HT + CMT + RDT
24	2021-5	60	C	1.3 cm*1.2 cm	Lower outer of right side	2.5*2.0*1.5 cm	3.0 cm*3.0*2.5 cm (AICAP)	135	13	No	HT + CMT + RDT + TT
25	2021-8	56	B	2.5 cm*1.7 cm	Lateral outer of right side	3.0*3.0*2.0 cm	6.0 cm*4.0*2.5 cm (LICAP)	145	10	No	CMT + RDT + TT
26	2021-10	57	B	1.5 cm*1.2 cm	Lateral inner of left side	2.5*2.0*1.5 cm	5.0*2.0*1.0 cm (AICAP)	108	8	No	HT + CMT + RDT
27	2021-5	47	B	2.0 cm*1.7 cm	Upper outer of right side	3.0*2.0*2.0 cm	6.0*4.0*2.0 cm (LTAP)	113	13	No	CMT + RDT + TT
28	2021-8	37	B	1.5*1.7 cm	Upper outer of left side	3.0*2.0*1.5 cm	4.0*3.0*3.2 cm (LTAP)	115	10	No	CMT + RDT
29	2022-3	38	A	2.0 cm*1.3 cm	Lower inner of left side	3.0*2.0*1.5 cm	6.0*3.0*2.0 cm (AICAP)	100	3	No	HT + CMT + RDT
30	2021-12	53	A	1.5 cm*2.2 cm	Upper outer of right side	3.0*2.0*1.5 cm	3.5*3.0*2.5 cm (LICAP)	104	6	No	HT + CMT + RDT

Three cases had TDAP applied to repair the tumour defects in the lateral or inner upper quadrant; in nine cases, LICAP flaps were applied to repair lateral or central breast area; eleven had AICAP applied for lower or inner upper quadrant, in seven cases, lateral tissue flap adipofascia with skin de-epithelialization or just separation of the subcutaneous tissue with no skin were utilized. *y* years old, *min* minute, *mths* months, *TDAP* thoracodorsal artery perforator flap, *LICAP* lateral intercostal artery perforator flap, *AICAP* anterior intercostal artery perforator flap, *LTAP* lateral thoracic artery perforator flap, *HT* hormone therapy, *CMT* chemotherapy, *TT* target therapy, *RDT* radiation therapy

All cases of invasive ductal carcinoma were diagnosed by preoperative crude needle biopsy, the tumour diameter was 1.5 to 4.2 cm, and 10 patients underwent neoadjuvant chemotherapy. The postoperative tumour stages were as follows: 6 in stage I, 15 in stage IIA, 8 in stage II B, and 2 in stage IIIA. The average resection area was 4.5*3.4*2.6 cm (2.5–6.0*5.0–2.5*1.5–3.5 cm). Size A, B, and C cups were covered, and there were mainly medium-small cups (A cup 7 cups, B cup 17 cups, C cup 6 cases). The tumours were located as follows: external quadrant (20 cases) and internal quadrant (10 cases). Lateral thoracic and abdominal perforator flaps, such as the anterior intercostal artery perforator flap (AICAP), lateral intercostal artery perforator flap (LICAP), thoracodorsal artery perforator flap (TDAP), or lateral thoracic artery perforator flap (LTAP), were applied for every site with a defect. When the tumour was located in the lateral breast (outer upper or outer lower quadrant), the lateral chest wall flap, thoracic dorsal artery perforator flap, or the intercostal artery side could be used. When the tumour was located at the lower pole or upper part of the breast, the intercostal artery anterior perforator flap or the fascia tissue flap above the rectus abdominis could be used. All patients signed an informed consent form before surgery after being informed of the surgical risks. Patient satisfaction was assessed at one month, three months, and six months after surgery.

## Operation process

### Preoperative preparation

(1) For all patients, a preoperative discussion was performed among the operation group, and these discussions included discussing nursing requirements for preoperative preparation (physical and psychological preparation, including skin preparation in the surgical area). (2) Confirmed implementation process of preoperative vascular positioning: Colour Doppler ultrasound physicians should have accepted positioning training on the vascular pedicles of the chest wall in advance. Colour Doppler was routinely applied to label the main vessels of the flap by ultrasound. (3) The operating surgeon drew the estimated resection range and the possible repair plan. The thickness of the flap was also estimated by the pinch test and was the approximate estimate of the effect of the flap filling back to the defect. Advanced rehearsal was sometimes required for some specific operations. (4) The surgical body position was confirmed during the operation, and the instrument nurse was informed to prepare a vascular identification band. The postoperative nursing requirements were formulated (including monitoring of the flap blood circulation in both the arteries and veins, observation of the drainage tube, and requirements for dressing or wearing chest tightness). The following data were routinely recorded before surgery: breast size, tumour size, location, and estimated defect volume.

### Operation implementation process

(1) The incision selection depended on the location of the mass and the possible location of the flap before surgery. Properly para-areola, lateral chest wall (which can be covered by a bra), or inframammary fold wall could be considered. (2) Completed conventional breast conservation resection: Based on the principle of tumour safety, the tumour with tissue edges more than 1 cm in large lesions was removed, and 4–6 margins were cut in the upper, lower, inner, and external directions. Sentinel node biopsy or conventional lymph node dissection was implemented, and the skin covering the tumour was removed at the same time if necessary. (3) The flap should be designed in a specific position (supine or lateral position, arm abduction, raised with small sterile pads outside the chest and underarms), adapting to the location of potential perforators. If no perforators were found before surgery, more small perforators could usually be found during donor area exploration. Peri-mammary artery perforator flaps could cover the repair of different quadrant defects. However, in the actual election of the perforator flap, the closest pedicle to the breast defect would always be applied so that the flap had enough mobility to transfer and fill the breast defect. When separating the flap, it was necessary to follow the dermatoglyphics away from the main perforator vessel of the flap and adopt a wedge resection method to separate the fat. The subcutaneous capillary network in the fascial tissue should be retained as much as possible, and more adipose tissue should be obtained to meet the filling needs. It was always necessary to use a blood vessel identification belt to pay attention to the protection of the pedicle vessels. When grabbing the flap, attention should also be paid to the length and width ratio. As long as the perforating vessel blood supply is excellent, the length and width ratio can appropriately exceed 3:1.

### Repair of the breast defect

**Fig. 1:**
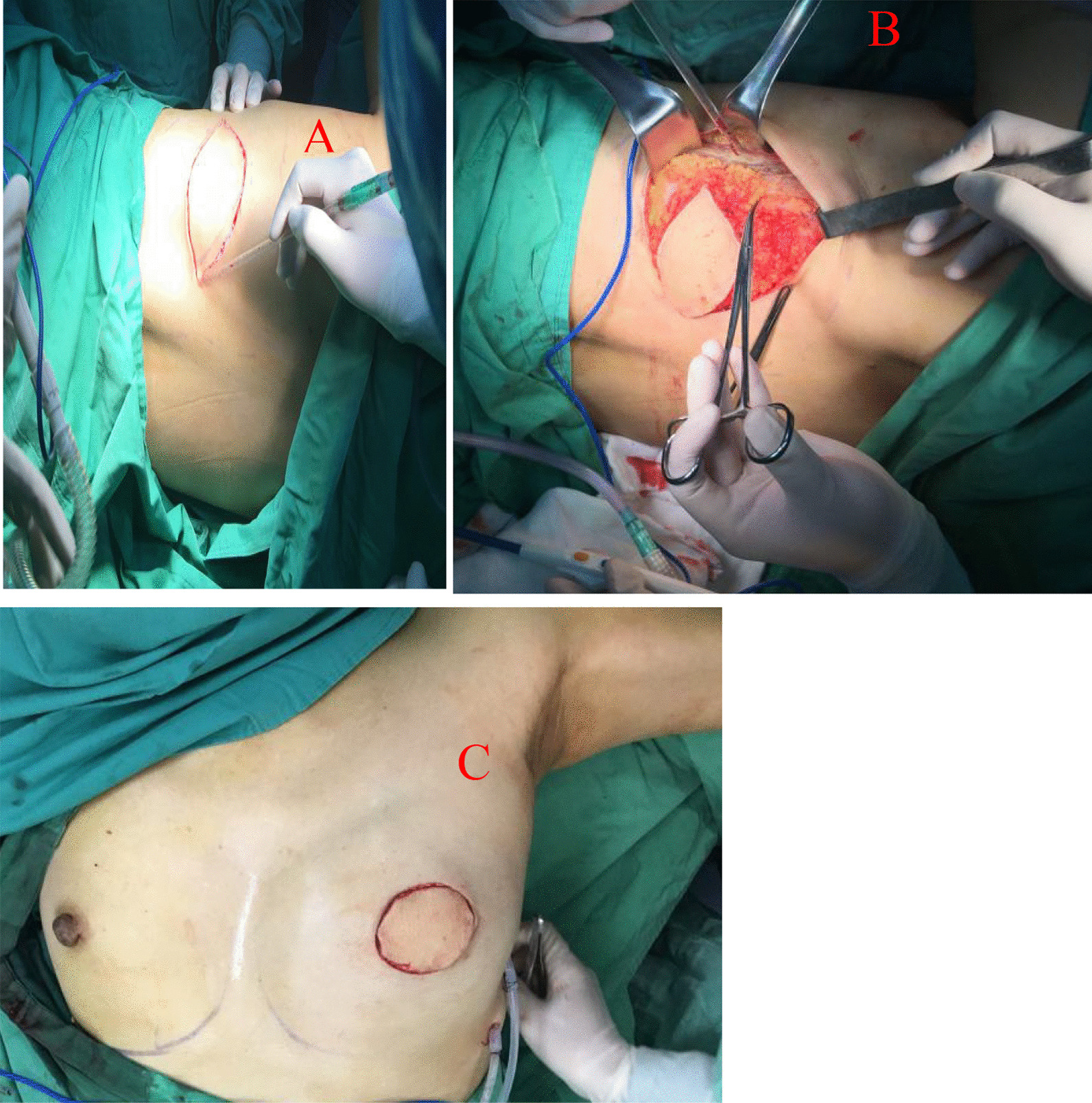
TDAP—**A** Begin with the outline preoperative. **B** Separation of the flap. **C** Filling the defect

**Fig. 2:**
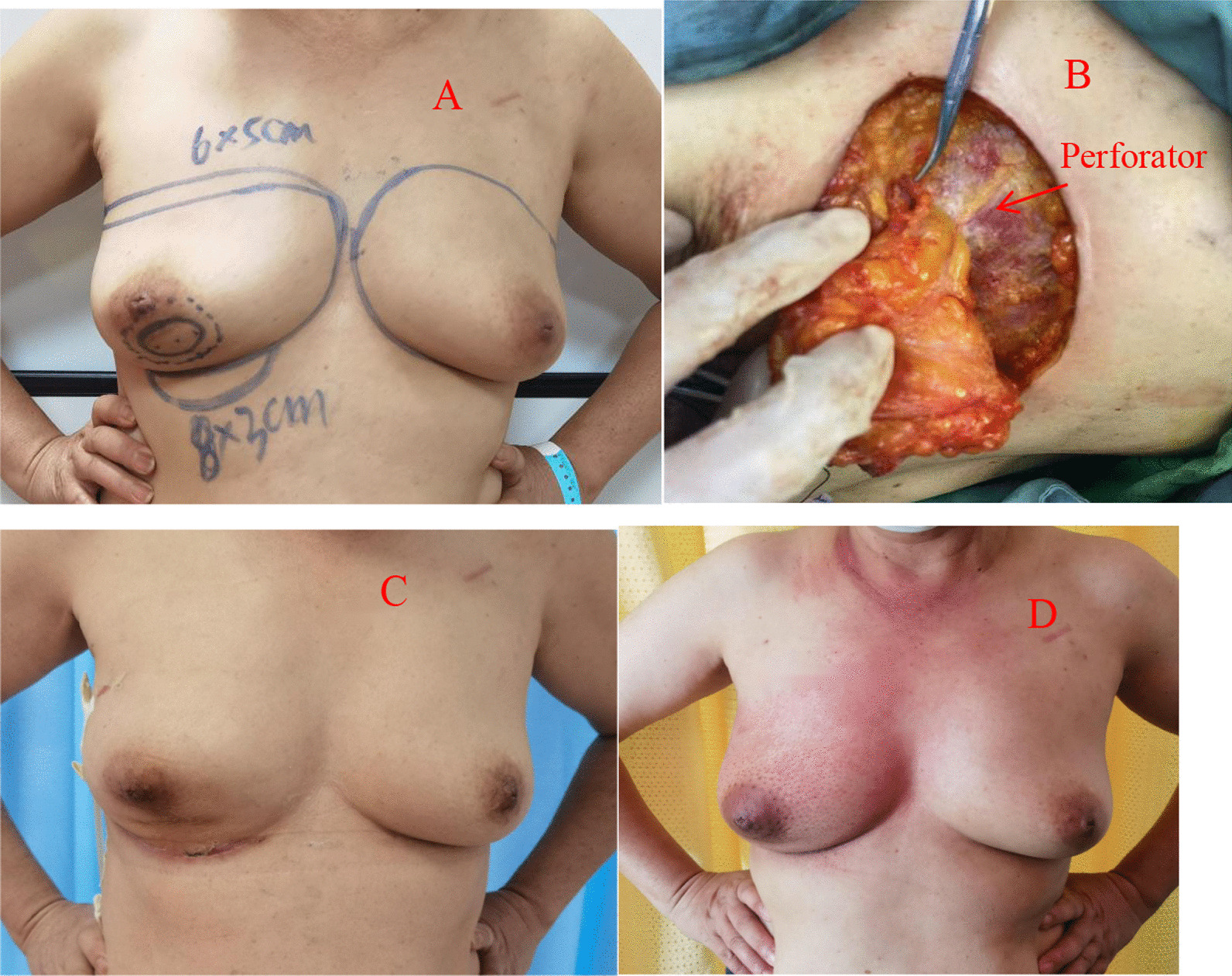
AICAP—**A** Marked whole breast outline and the precise location of the tumour (the patient underwent neoadjuvant chemotherapy for 8 cycles). **B** One of the perforators found intraoperatively. **C** Two weeks after the operation. **D** Three months after the operation (the patient had finished radiation a month prior)

**Fig. 3:**
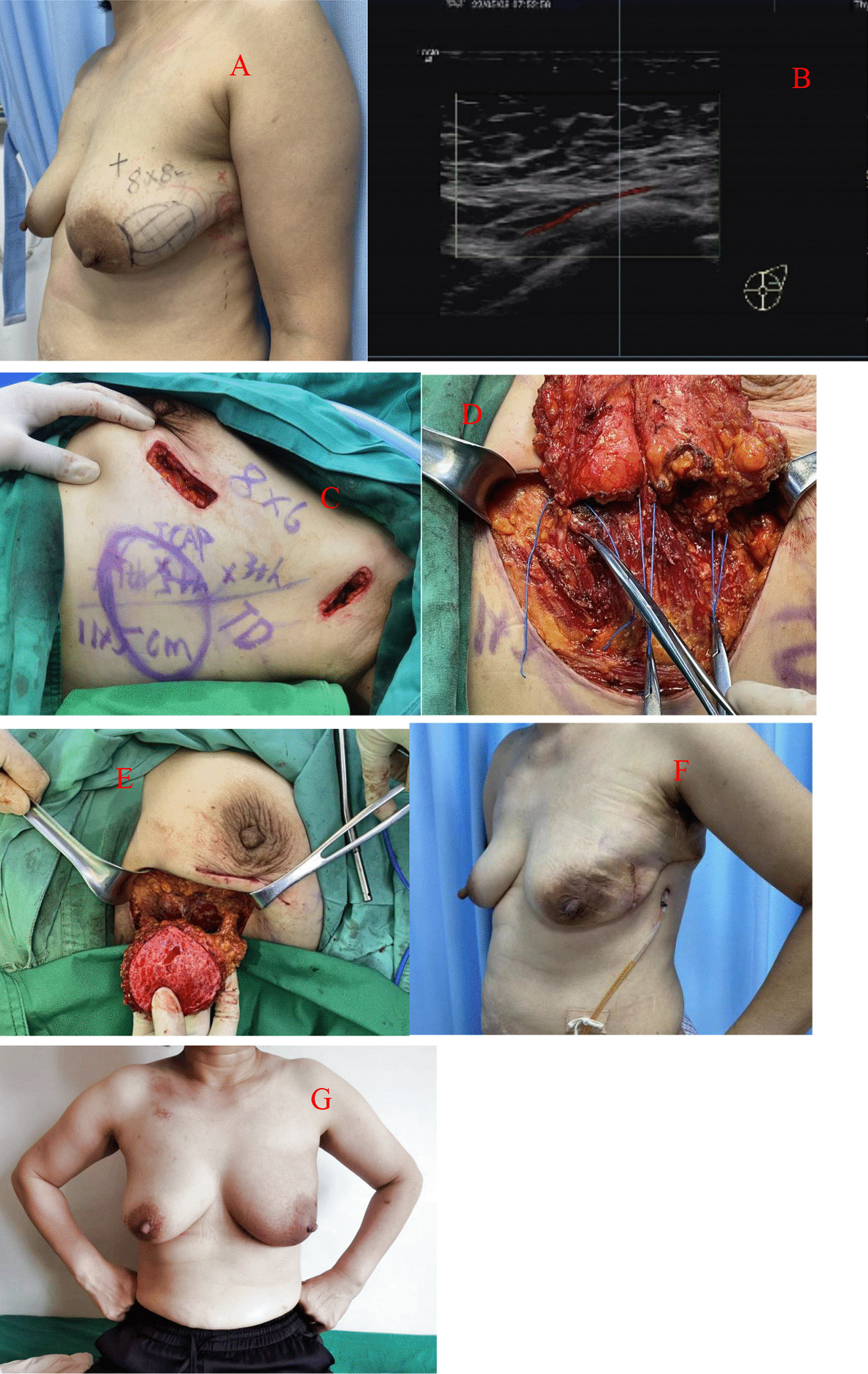
LICAP—**A** The precise location of the tumour was outlined. **B** The 5th lateral intercostal artery perforator in a colour Doppler ultrasound image. **C** Designed donor site to repair the defect. **D** The 4th, 5th, and 6th intercostal artery perforators were found intraoperatively, and a vessel belt was used to protect them months after the operation. **E** De-epithelialization of the flap. **F** Two weeks after the operation, the patient experienced fat liquefaction. **G** Three months after the operation

**Fig. 4:**
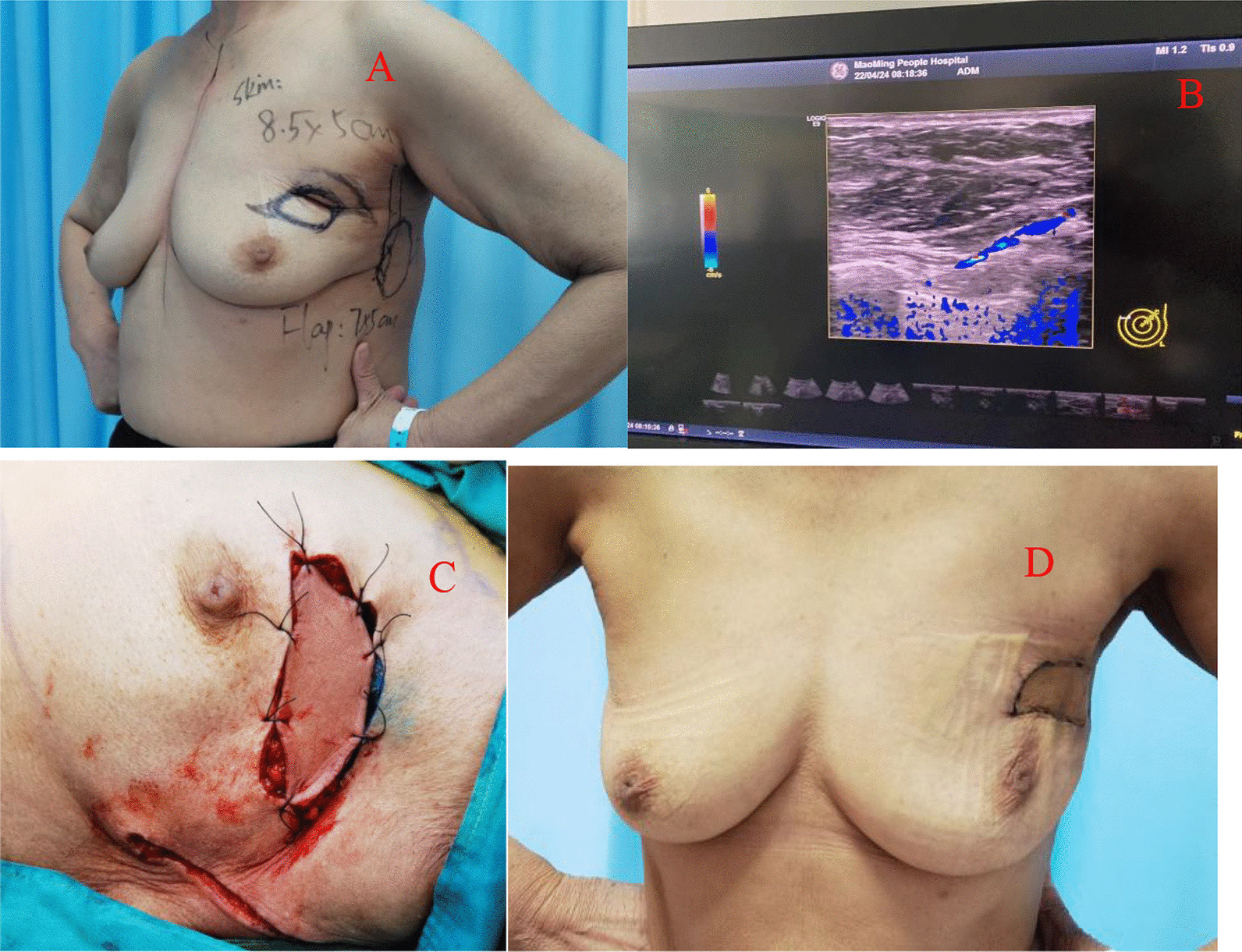
LICAP—**A** Designed resection plan and reconstruction flap preoperatively. **B** The 4th lateral intercostal artery perforator in a colour Doppler ultrasound image. **C** Filling the defect. **D** Three months after the operation

**Fig. 5:**
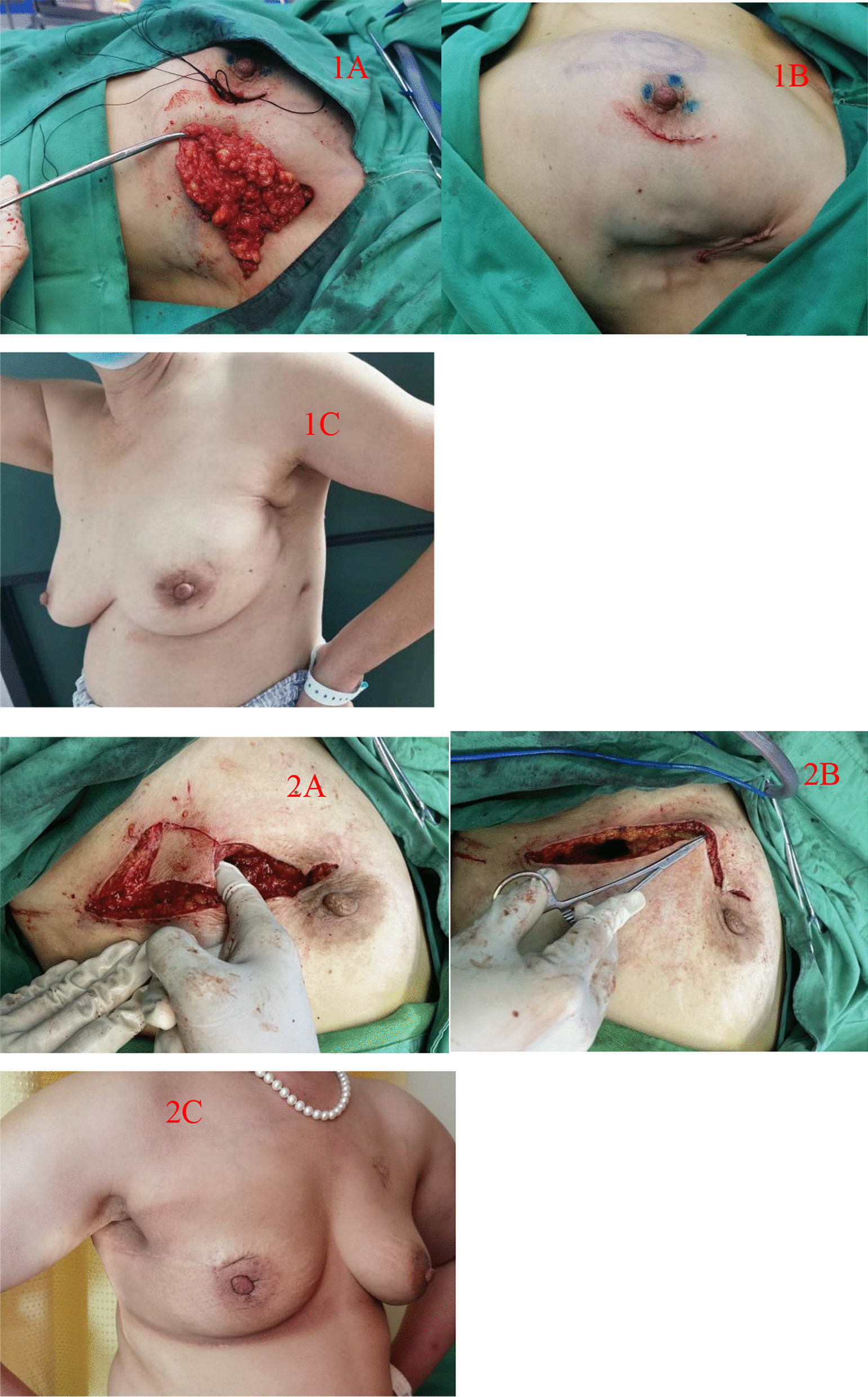
LTAP—**1A** Separation of the lateral thoracic adipofascia. **1B** A picture after finishing suturing. **1C** Three months after the operation; **2A** Design of a “Z”-shaped tissue flap. **2B** De-epithelialization and filling of the defect. **2C ** Three months after the operation

1. Application of a TDAP (Thoracodorsal artery perforator flap): If the thoracodorsal flap perforator was chosen, the anterior boundary was at the lateral margin of the pectoralis major muscle, the posterior boundary was at the anterior edge of the latissimus dorsi muscle, the upper boundary was at the thoracodorsal vessel bifurcation of the axillary vessels, and the lower boundary could reach the umbilical level due to sufficient bare perforations of the thoracic dorsal artery branch [8]. This flap could be designed to have a more extensive range of motion than other regional flaps. Therefore, it could be used for defects in various quadrant areas of the breast. Of course, in the actual implementation process, if the local area flap could meet the requirements, there was no need to use this flap. It was also selected according to the different factors considered by each surgeon or the actual situation during the operation. (group Fig. 1).
2. Application of an AICAP (Anterior intercostal artery perforator flap): If the defect was located in the lower or inner upper quadrant, the intercostal artery anterior perforator flap could be chosen, the upper side was the inframammary fold, and an appropriate width should be considered according to the size of the defect. In the process of obtaining the flap, the adipofascia tissue above the rectus abdominis muscle could be simultaneously obtained as much as possible, and part of the rectus abdominis sheath tissue could be carried at the same time to stabilize the flap blood supply. Flap acquisition was not just for filling defects. The dermal fascia flap with the removed epidermis could be used to further reduce the possibility of fat liquefaction or necrosis of the postoperative tissue flap [9]. (see group Fig. 2)
3. Application of the LICAP (lateral intercostal artery perforator flap): When the defect was in the outer lower quadrant, the outer upper quadrant, or the central area, an intercostal artery perforator flap was designed. According to the results of nearly 200 autopsy cases performed by Hamdi et al., the perforators are located approximately between the 3rd–8th intercostal space. The more concentrated ones are between the fifth and seventh ribs within approximately 2.5–3.5 cm from the anterior edge of the anterior serratus muscle [10]. In addition to conventional empirical body surface localization, vascular colour ultrasound was used to clarify the actual location of the puncture branch before the operation. The upper boundary was usually defined at the subthoracic and dorsal vascular bifurcation of the axillary vessels, the lower boundary could be reached below the inframammary fold, the anterior boundary was at the lateral margin of the pectoralis major muscle, the posterior boundary was at the anterior edge of the latissimus dorsi, and the incision sequence was made from far to near in a clockwise sequence with posterior-lower–upper-anterior. The flap was also rotated 180 degrees to the defect, and if the defect area was too far that the flap could not reach, such as the inner upper or upper quadrant, volume displacement technology was applied at the same time in a strategy with A tissue moving to tissue B and tissue B moving to tissue C, ensuring no torsion in the pedicle vessel. (see group Figs. 3 and 4)
4. Application of the LTAP (lateral thoracic artery perforator flap): When the defect is located on the lateral side of the breast, and if the subcutaneous tissue of the lateral chest wall is relatively plump, perforators in the lateral chest wall near the armpit can be located before surgery [11, 12], and the flap might include the lateral thoracic artery (lateral thoracic branch), intercostal artery and serratus anterior branch of the dorsal thoracic artery. There is no need to expose all perforators during manipulation, and attention must be paid to retaining the vascular plexus of the fascia flap, including the subfascial, middle, and upper vascular networks. A rough tissue flap area was marked according to the defect size during the operation. It was recommended to use a sharp scalpel to detach the fascia. The tissue flap should contain the subcutaneous vascular network and superficial and deep fascia with no skin. Attention was given to the level of the peeling plane to ensure that the flap was smooth and maintained the same thickness and depth. Generally, to maintain the continuity of the vascular network, the tissue flap anterior boundary should be in the thoracic lateral margin, the posterior boundary was in the latissimus doralis anterior, the upper edge was in the axillary vessels below the branch intersection, and the lower boundary could reach an umbilical level with a counterclockwise incision, from far to near. The tissue flap was fixed with a 2-0 absorbable suture when an arbitrary LTAP flap was transferred to the breast defect (as shown in Group Fig. 4). For example, when the defect is located in the upper quadrant of the breast and the patient’s lateral chest wall is relatively plump, a "Z"-shaped flap with de-epithelialization can also be designed. (see group Fig. 5)

## Cosmetic evaluation

To assess patient satisfaction with the general and aesthetic outcomes of the oncological surgery, we used the extracted version of the BREAST-Q version 2.0, Breast Conserving Therapy Module Preoperative and Postoperative Scales [13, 14] to form a new breast reconstruction satisfaction questionnaire, which was administered at one month, three months, and six months after surgery. Each question was scored on a 5-point scale, ranging from "very satisfied [5]" to "not satisfied [1]" (see Table 2). We also compared patients' satisfaction with different flaps. SPSS 21.0 statistical software was used for the statistical analysis of the data. The chi-square test was used to compare the differences between the groups. All statistical data were statistically significant at P < 0.05.

**Table 2:** Breast reconstruction satisfaction chart

Breast reconstruction satisfaction chart					
Question	Very satisfied		→		Not satisfied
Q1. Confidence	5	4	3	2	1
Q2. Impact of sexual life	5	4	3	2	1
Q3. Outlook in the mirror	5	4	3	2	1
Q4. Dressing feelings	5	4	3	2	1
Q5. Appearance on the breast-preserving side	5	4	3	2	1
Q6. Bilateral breast symmetry	5	4	3	2	1
Q7. Discomfort in the surgical site	5	4	3	2	1
Q8. Healing time	5	4	3	2	1
Q9. Scar	5	4	3	2	1
Q10. Expected outcome	5	4	3	2	1

The BREAST-Q breast reconstruction satisfaction questionnaire was performed at 1month, 3 months, and 6 months, respectively after surgery. Each question was scored on a 5-point scale, ranging from "very satisfied [5]" to "not satisfied [1]"

## Results

A total of 30 patients received volume replacement techniques from 2021 to 2022 after BCS. The mean age was 54 years (33–65 years), and the mean follow-up interval was 9.3 months (3–17 months). A preoperative search for vascular perforations in all cases was performed by Doppler ultrasound. In the first patient, the vascular perforations were attempted to be sought out through CTA. However, it was difficult to accurately complete the identification of the finer perforators both in CTA and body surface positioning. Given the increased cost of the inspection and the problem of radiological damage, in the operation group, CTA was abandoned for localization. Vascular colour ultrasound identification proceeded routinely in all subsequent patients. Mostly, the required perforators could be found. If perforators were unable to be found or if other unplanned available perforators were found, adjusting the original plastic surgery strategy could be managed. It is more challenging to find braces for AICAP because they are all thinner perforators. Therefore, careful fascial sharp separation from far to near should be implemented. It was important to protect every tiny perforator before deciding the superior perforator when the blood supply situation of the flap was clear. In the search process of each vascular perforator, if a microscope or a head-wear microscope could be utilized, the efficiency of perforator finding could be improved, and the quality of perforator blood transport could be guaranteed. The identification and application of the perforator can only be realized through the preoperative positioning of the vascular perforator and with a careful intraoperative operation due to the lack of a valid microscope. Usually, one or more perforators can be found and used per flap, but it is not recommended to use them all because they will potentially affect flap rotation. The average surgical time (including tumour resection and reconstruction) was 142 min (100–250 min). The time to flap cutting, peeling, and remodelling averaged 45 min (30–60 min). The surgeries are not easy when surgeons begin this technique, but surgical time is eventually saved after the learning curve was established (not until approximately case number 8).

In three cases, a TDAP was applied to repair tumour defects in the lateral or inner upper quadrant, LICAP flaps were applied in nine cases to repair the lateral or central breast area, an AICAP was applied in eleven cases for the lower or inner upper quadrant, and lateral tissue flaps adipofascia with skin de-epithelialization or just separation of subcutaneous tissue with no skin was applied in seven cases. No wound infection occurred, but one AICAP flap developed fat necrosis, which recovered after local treatment with conventional suction flow. Local tissue stiffness in the area was left in the short term in the following half year, but the situation gradually decreased after breast shaping and active fat hypertrophy. One patient with an LICAP developed venous congestion on the first day after surgery. The acupuncture flap test showed good blood supply; the operation group considered it to come with poor venous reflux because of the relative sagging of the patient's breasts; and the dressing could not support the breast effectively, which led to the excessive pulling of the pedicle blood vessels. In addition, the simultaneous placement of the drain was located mainly at the bottom of the flap, the flap was located below the nipple-areola, and the effusion below the nipple-areola failed to drain smoothly. The increased effusion further aggravated the flap tension. However, after specific monitoring by the nursing team and the improvement of the increased flap tension caused above, the venous congestion gradually improved, and the flap blood circulation returned to normal 4–5 days after surgery.

**Fig. 6:**
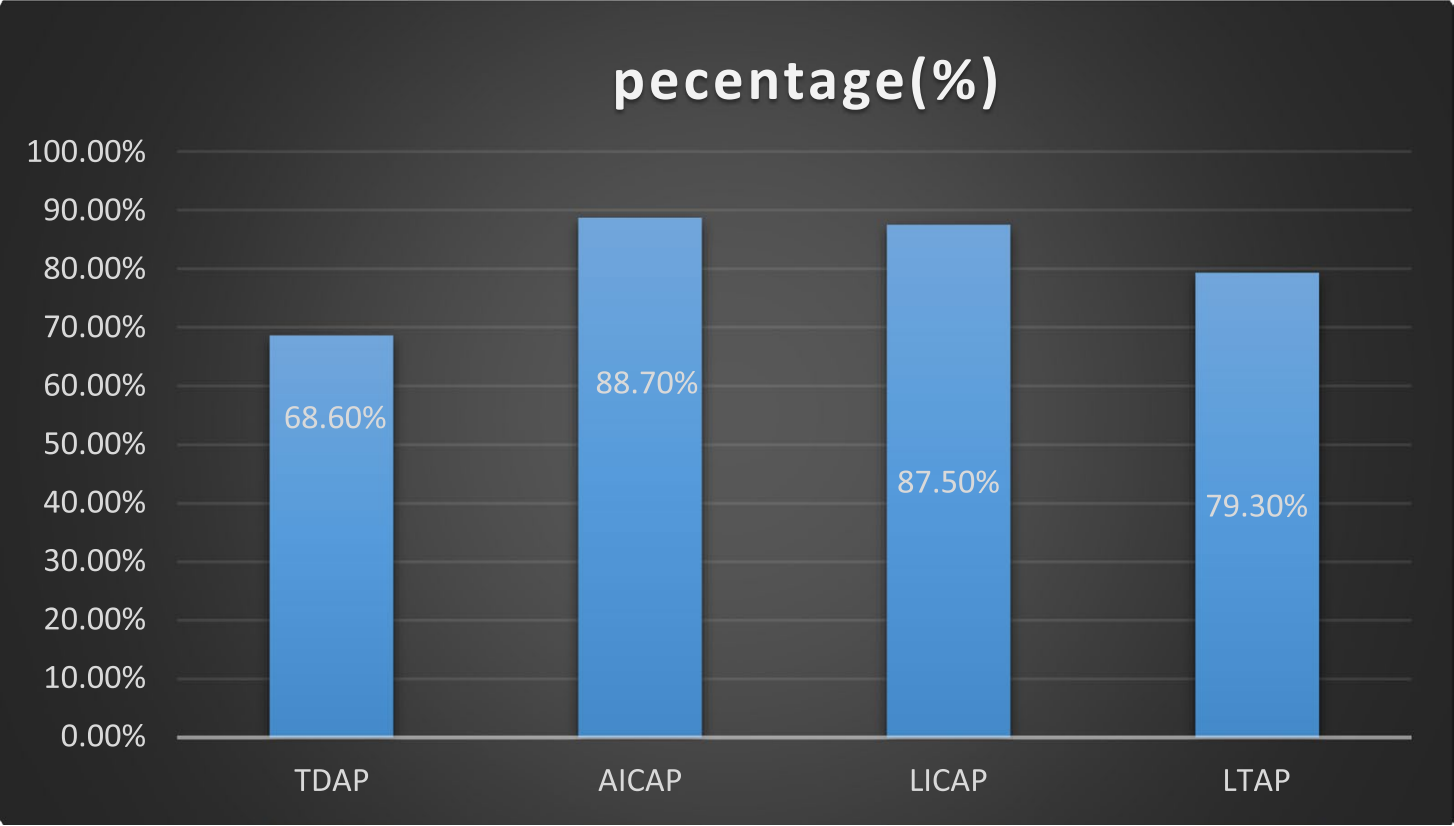
The results of the patient satisfaction surveys according to the various types of flaps. *AICAP* anterior intercostal artery perforator flap, *LICAP* lateral intercostal artery perforator flap, *TDAP* thoracodorsal artery perforator flap, *LTAP* lateral thoracic artery perforator flap

The patient satisfaction survey results are presented in Tables 3, 4, and Fig. 6. The mean score of patient satisfaction with bilateral breast appearance was 85.3 in size and symmetry, and the mean confidence in sexual life was 80.8 postoperation. There were no significant differences in holistic satisfaction between the preoperative and postoperative scores. The uncomfortable sensations of the operative region improved over time. Patients were satisfied with the reconstruction technique of peri-mammary artery perforator flaps after breast-conserving, and the satisfaction of AICAP and LICAP was higher. These results suggest that most patients regard this surgical outcome as satisfactory and that it is worthwhile to promote these surgical techniques.

**Table 3:** Breast-conserving patient satisfaction

	Satisfaction degree		
	Postoperation	Preoperation	p value
*Dressing satisfaction*			0.536
Confidence			
Satisfied	27 (90%)	28 (93.3%)	0.640
Not satisfied	3 (10%)	2 (6.7%)	
Dressing feeling			
Satisfied	26 (86.7%)	29 (96.7%)	0.161
Not satisfied	4 (13.3%)	1 (3.3%)	
*Sexual life*			
Sexual life			0.278
Satisfied	24 (80%)	27 (90%)	
Not satisfied	6 (20%)	3 (10%)	
*Appearance*			0.839
Outlook in the mirror			1.000
Satisfied	26 (86.7%)	26 (86.7%)	
Not satisfied	4 (13.3%)	4 (13.3%)	
Appearance of the surgery breast			0.301
Satisfied	27 (90%)	29 (96.7)	
Not satisfied	3 (10%)	1 (3.3%)	
Symmetry			0.317
Satisfied	23 (76.7%)	26 (86.7%)	
Not satisfied	7 (23.3%)	4 (13.3%)

**Table 4:** Satisfaction score of breast-conserving area

	Satisfaction degree at different times after surgery			
	1 month	3 months	6 months	p value
Feelings in the surgical site				0.109
Satisfied	18 (60%)	23 (76.7%)	25 (83.3%)	
Not satisfied	12 (40%)	7 (23.3%)	5 (16.7%)	
Recovery state				0.862
Recovery time				0.960
Satisfied	24 (80%)	24 (80%)	24 (80%)	
Not satisfied	6 (20%)	6 (20%)	6 (20%)	
Scar				0.638
Satisfied	22 (73.3%)	25 (83.3%)	23 (76.7%)	
Not satisfied	8 (26.7%)	5 (16.7%)	7 (23.3%)

## Discussion

In 1981, Veronesi et al. [2] reported no significant difference in the survival rate of breast preservation quadrant resection plus radiotherapy and modified radical. In 1985, numerous clinical studies showed that breast-conserving surgery and postoperative radiotherapy had the same survival rate as mastectomy [15, 16], and the quality of life in patients who underwent breast preservation surgery was significantly higher than that in patients who underwent mastectomy [17, 18]. Therefore, breast-conserving surgery (BCS) has been accepted as the primary treatment modality for patients with stage I and II breast cancer. However, a breast deformity caused by BCS is the primary defect [3–5]. In recent years, the breast plastic technique has been used in breast surgery more frequently, leading to the emergence of a concept called oncoplastic breast-conserving surgery (OBCS) [19, 20]. For patients with < 20% of their breasts with breast defects or patients with a generally large breast volume, applying the volume-based displacement technology would come with a good effect, but for those patients with breast defects reaching more than 20%, these defects always need to be repaired using autologous tissue, such as filling the defects with a musculocutaneous flap or a local flap (lateral chest wall flap or a thoracoabdominal flap) by rotation, shifting and remodelling the breast appearance [21–25].

As early as 2004, Hamdi et al. and Lopez et al. successively reported the application of thoracic and dorsal artery perforator flaps (TDAP), ICAP and AICAP without sacrificing muscle in breast-conserving surgery. They obtained the perforator of the corresponding regional artery intraoperatively mainly through preoperative colour ultrasound positioning and the utilization of plastic surgery techniques [26–28]. However, the application of this kind of perforator flap technique for breast reconstruction is still not quite widespread in China. The data from a study on breast-conserving plastic surgery in 110 centres from China suggested that the volume displacement technique was significantly higher than the volume replacement technique (60% vs. 15.2%). Therefore, there is an increasingly emerging need to obtain the indications, technical reference, design concept, and patient satisfaction data to implement positioning accuracy and a strategy refinement part of breast reconstruction technology in clinical practice.

In this study, in the operation group, different perforator flaps were selected in consideration of the tumour location, tumour size, and size of the removed breast tissue. For cases with resected tissue > 20%, for example, tumours located in the lateral side of the breast, a thoracodorsal artery perforator flap or a lateral intercostal artery preperforator flap could be selected. In contrast, the anterior intercostal artery perforator flap could be used for tumours located in the lower breast pole. In addition, the inner upper quadrant has been a complex area to repair defects but could be adopted as a forwards propulsion technique. The adjacent tissue flap could be dissociated and pushed to the inner upper quadrant, and then the anterior intercostal artery with or without adipofascia tissue above the rectus abdominis muscle was free to fill the inner lower defect because of its limited perforator mobility. When encountering a more plump body shape or entirely subcutaneous tissue at the armpit or lateral chest wall, the defects in the upper breast quadrant could be filled directly by designing a Z-shaped flap or a lateral adipofascia tissue flap. No flap necrosis was observed to require secondary debridement surgery. The main postoperative complication observed was inevitable fat necrosis, although it did not cause necrosis of the whole flap necrosis nor cause flap loss. However, even part of the fat necrosis could also cause short-term stiffness in the reconstruction part. When tumour skin invasion is suspected, the flap with good blood circulation could directly be used to fill the defect as a de-epithelialization defect, and this retains a particular area of skin graft in the flap for repair and monitoring. Drainage tubes were retained for 5–7 days, and the patients were followed-up for one month, three months, and six months to observe the flap shape status and take photos to record and conduct the BREAST-Q survey score to generate a patient chart, which consisted of all the patients’ information preoperation and postoperation. Overall, there were no significant changes in the baseline preoperation and postoperative BREAST-Q scores for the 30 patients. The intercostal perforator flap (AICAP, LICAP) had the highest scores for breast shape (mainly with an A cup or B cup breast), donor area scar satisfaction, and surgical area discomfort. In general, patients were more satisfied with AICAP and LICAP when investigating the satisfaction of different flaps, which suggest that intercostal perforator flaps are suitable for partial breast reconstruction in patients with small-medium-sized breasts without a negative impact on patient satisfaction. These results suggest that most patients regard this surgical outcome as satisfactory, and it is worthwhile to promote these surgical techniques.

Each patient had vascular positioning performed by vascular colour ultrasound to find the nearest perforators to the defect site, and a right flap was designed to fill in the defect during the operation according to its designed direction. In this study, lower scores were associated with bilateral breast symmetry, which might relate to the intraoperative inability to accurately measure the volume size of the defect gland and predict the long-term effect at the lay of three-dimensional space. When the gland is moved and the flap tissue repair is mainly fascia or adipose tissue, there are some differences in the specific volume saturation between these two kinds of tissues. Even though they are autologous, some of the filled adipose tissue inevitably exhibits liquefaction necrosis simultaneously, yet patients with high requirements can obtain their appearance satisfaction through autologous fat granule filling.

The deficiency of this study was the inability to measure the size of the breast defect and the possible shape. Some previous studies measured the preoperative size ratio of tumour size and breast volume to outline the defect through MRI, and the measured volume could be used to design enough flaps for repair. This technique has been reported in many kinds of literature of retrospective studies, but the reference value for its effect is relatively poor. Artificial intelligence technology might help to calculate the estimation of the gland defects, flap position, design, flap thickness, donor shape and even the distance of the flap moving to the defect. More data can be specific to provide comprehensive data to accurately implement oncoplastic breast surgery, ultimately achieving the maximization of bilateral breast symmetry.

## Conclusion

The centre summarizes the limited data above and provides the basic pattern of the technique but does not represent the highest level or the most detailed specific surgical steps. All in all, patients were satisfied with the reconstruction technique of peri-mammary artery perforator flaps after breast-conserving according to this study, and the satisfaction of AICAP and LICAP was higher, this technique is suitable for partial breast reconstruction in A-cup or B-cup breasts, and it does not appear to negatively impact patient satisfaction. There is a great chance for knowledge exchange in the centre, which can cause a revolution of knowledge.

## Data Availability

All data generated or analysed during this study are included in this published article.
